# What drives the excess of physical exercise observed in patients with anorexia nervosa?

**DOI:** 10.1192/j.eurpsy.2021.947

**Published:** 2021-08-13

**Authors:** L. Di Lodovico, H. Hatteea, C. Couton, P. Duriez, J. Treasure, P. Gorwood

**Affiliations:** 1 Hopital Sainte-anne Ghu Paris Psychiatrie Et Neurosciences, Université Paris Descartes, Paris, France; 2 Groupement Hospitalier Universitaire (ghu) Paris Psychiatry And Neuroscience, Sainte-Anne Hospital, Paris, France; 3 Institute Of Psychiatry, King’s College, London, United Kingdom

**Keywords:** physical exercise, anorexia nervosa, endophenotype, emotions

## Abstract

**Introduction:**

Anorexia Nervosa (AN) is a severe mental illness characterized by weight reducing strategies such as food restriction, purging behaviours and excessive physical exercise. The persistence of physical exercise despite underweight and its maintaining factors are poorly understood.

**Objectives:**

The aim of this study is to explore the attitudes towards physical exercise and its effects on mood, body image perception and cognitive functioning in patients with AN, and to assess if these effects are associated with trait, or state.

**Methods:**

Eighty-eight patients with AN, 30 unaffected relatives and 89 healthy controls were compared about their attitudes towards three aspects of physical exercise, namely the Exercise Dependence Scale (EDS), the Godin Leisure Time Exercise Questionnaire (GLTEQ) and a standardized effort test. Evaluations of positive and negative affects, cognitive rigidity and body image distortion were repeated at baseline and after the effort test to assess for correlations between the exercise measures and exercise-induced modifications in the three groups.

**Results:**

Patients with AN showed higher scores on the EDS, the GLTEQ and used more effort in the standardized effort test (p<.05). These three aspects of physical exercise correlated with baseline negative emotions (p<.01). AN patients and unaffected relatives, but not controls, showed a marked emotional improvement after physical exercise (p<.01).

**Conclusions:**

Excessive physical exercise seems a trait-associated feature of AN, driven by a state-related effect of physical exercise on emotional wellbeing. The mood-related drive for physical exercise has the characteristics of an endophenotype in the patients of the present sample.

